# Discovery of KDM5D as a novel biomarker for traumatic brain injury identified through bioinformatics analysis

**DOI:** 10.3389/fimmu.2025.1538561

**Published:** 2025-03-24

**Authors:** Dengfeng Ding, Mengzhe Yang, Xinou Zheng, Ming Zhao

**Affiliations:** ^1^ Medical Innovation Research Department, Chinese PLA General Hospital, Beijing, China; ^2^ Beijing Anzhen Hospital, Capital Medical University; Key Laboratory of Remodeling-related Cardiovascular Diseases, Ministry of Education, Beijing Collaborative Innovation Center for Cardiovascular Disorders, Beijing Institute of Heart, Lung and Blood Vessel Diseases, Beijing, China; ^3^ Department of Neurosurgery, First Medical Center, Chinese PLA General Hospital, Beijing, China

**Keywords:** traumatic brain injury, KDM5D, biomarker, bioinformatics analysis, neuroinflammation

## Abstract

**Background and aim:**

Traumatic brain injury (TBI) poses a significant burden on the global economy due to its poor treatment and prognosis. Current TBI markers do not comprehensively reflect the disease status. Therefore, identifying more meaningful biomarkers is beneficial for improving the prognosis and clinical treatment of TBI patients.

**Methods:**

The gene expression profile of TBI was obtained from the Gene Expression Omnibus (GEO) database. Differentially expressed genes (DEGs) were subjected to enrichment analysis, and key potential genes were identified through the protein–protein interaction network and cytoHubba modules. ROC curves were used to construct diagnostic models for hub genes. Immunofluorescence experiments were conducted to detect the expression of candidate biomarkers in TBI rat models. Finally, we investigated the expression of TBI biomarkers in normal human organs and pan-cancer tumor tissues, and evaluated their correlation with immune infiltration in different tumors.

**Results:**

A total of 44 DEGs were identified across four brain regions of TBI patients. Enrichment analysis revealed that these genes were primarily involved in intracellular and cell signal transduction pathways. Furthermore, three hub genes- RPS4Y1, KDM5D and NLGN4Y-were identified through different module analysis. The ROC curve diagnostic model also confirmed that these genes also have high diagnostic value in serum. Subsequently, the presence of Kdm5d was detected in the brain tissue of TBI rats through immunofluorescence experiments. Compared to normal rats, Kdm5d expression increased in the cortical area of ​​TBI rats, with no significant change in the hippocampus area, aligning with observations in TBI patients. Immune infiltration analysis demonstrated changes in immune cell subsets in HIP and PCx, revealing that plasma cells and CD8 T cells were lowly expressed in TBI (HIP) and while neutrophils was under-expressed in TBI (PCx). Pan-cancer analysis indicated that KDM5D was significantly up-regulated in 23 cancers, down-regulated in 3 cancers, and significantly associated with immune infiltration in 10 cancers.

**Conclusion:**

Based on the results of bioinformatics analysis and animal experiments, KDM5D serves as a potential biomarker for the diagnosis and prognosis of TBI. Additionally, research on KDM5D may develop into new serum markers, providing new indicators for further clinical liquid biopsy and aiding in the prevention of both TBI and tumors to a certain extent.

## 1 Introduction

Traumatic brain injury (TBI) is defined as a condition that disrupts brain function or causes other brain lesions due to external physical forces (1). It is estimated that the annual incidence of TBI is 50 million cases worldwide, with roughly half of the population likely to experience a TBI in their lifetime. Additionally, morbidity and mortality rates are higher in low- and middle-income countries. TBI results in approximately US$400 billion in economic losses globally each year, accounting for 0.5% of the world’s gross product (2).

Currently, assessing the extent of injury and predicting outcome in TBI patients remains challenging. The Glasgow Coma Scale (GCS) and Abbreviated Impairment Scale (AIS) correlate with injury severity and provide prognostic power. However, these scales rely on the medical staff’s subjective interpretation of the patient’s injury and brain function, and their accuracy can be affected by factors such as age and other variables (3–5). Biomarkers offer an unbiased and independent assessment of TBI severity and patient prognosis. Studies had shown that glial fibrillary acidic protein (GFAP), S100β and soluble urokinase plasminogen activator receptor (suPAR) have been used to evaluate the survival rate of TBI patients (6–8). Additionally, tau protein and GFAP have been used to assess GCS scores at discharge and Glasgow Outcome Scale (GOS) scores at 6 or 12 months (6, 9). Ubiquitin C-terminal hydrolase (UCH-1), matrix metalloproteinase 9 (MMP9) and MMP2 are also believed to to be related to TBI severity (10, 11). Most of these biomarkers can be measured from plasma or serum samples, minimizing the challenges of cerebrospinal fluid (CSF) collection, but rapid turnaround remains a concern in the critical TBI settings.

With the rapid advancement of high-throughput technologies and bioinformatics, we can now screen biomarkers and therapeutic targets more efficiently. Previous bioinformatics studies have primarily identified biomarkers through differential gene and protein-protein interaction (PPI) network analysis (12). However, the accuracy of PPI network analysis has been questioned. With evolving analysis methods, tools like Cytoscape and its plugins are increasingly used to screen potential disease targets. Cytoscape is an open-source software that integrates biomolecular interaction networks with high-throughput expression data and other molecular states into a unified conceptual framework (13).

In this study, we first obtained the TBI microarray dataset from the Gene Expression Omnibus (GEO) public database to analyze and identify differentially expressed genes (DEGs). We then combined bioinformatics analysis with machine learning strategies to deeply screen and identify key features of TBI. Receiver operating characteristic (ROC) analysis was used to evaluate their diagnostic values. Recently, accumulating evidence suggests that immune and inflammatory mechanisms play crucial roles in preventing the pathogenesis of TBI (14, 15). Therefore, we aimed to explore the relationship between biomarker genes and immunity. We applied cell-type identification by estimating relative subsets of RNA transcript (CIBERSORT) to clarify the differences in immune infiltrates in TBI. Subsequently, we verified the expression of biomarkers in the brain tissue of TBI rat’s model. In recent years, pan-cancer analyses have enabled us to leverage human genome sequence data, along with a vast compendium of associated molecular and phenotypic features and functional genomic data from genome-scale expression and epigenomics. This helps us understand the impact of variants and elucidate the contribution of dysregulated genes to specific diseases and biological pathways in tissue contexts (16). Finally, we analyzed the expression and immune cell infiltration of KDM5D in various cancers, which may significantly enhance our understanding of the biological function of KDM5D. We propose that KDM5D can serve as a common biomarker for predicting and diagnosing TBI and pan cancer. However, this statement has certain flaws, such as distinguishing patients who suffer from both TBI and cancer, understanding the mechanism of KDM5D upregulation changes, and combining other biomarkers of these two diseases may be a more reliable clinical strategy.

In summary, this study demonstrates that KDM5D is a robust and feasible new biomarker for TBI, providing a potential target for the diagnosis, prevention, and treatment of TBI.

## 2 Materials and methods

### 2.1 Data acquisition and processing

The mRNA transcriptome profiles of patients with TBI were downloaded from the NCBI Gene Expression Omnibus public database. (GEO, https://www.ncbi.nlm.nih.gov/geo/). We selected GSE104687 as the dataset for TBI, which includes 376 samples collected from cortical grey (parietal and temporal), white matter (parietal) and hippocampus from a total of 107 brains, including 55 TBI patients and 52 No-TBI patients. GSE254880 was chosen as the validation set, consisting of neuronal exosomes samples from serum, with 8 control samples and 8 TBI patients. Data filtering and preparation were completed using the R program (version 4.2.1), and batch effect were eliminated. The basic information of all datasets was shown in **Table 1**.

**Table 1:** The details of datasets.

Series	Platforms	Samples	Tissue	Type
GSE104687	GPL16791	55 TBI patients and 52 No-TBI patients’ samples	brain samples	mRNA
GSE254880	GPL20301	8 control samples and 8 TBI patients	Serum samples	mRNA

### 2.2 Identification of differentially expressed genes

Limma (Linear Models for Microarray Data) is a differential expression screening method based on generalized linear models. Here we use the R software package limma (version 3.40.6) to conduct differential analysis and identify differences between comparison groups and the control group. Specifically, we obtained the expression profile dataset, performed multiple linear regression using the lmFit function, and computed moderated t-statistics, moderated F-statistic, and log-odds of differential expression using the eBays function via empirical Bayes moderation of the standard errors towards a common (17). Genes with log2FC (fold change)>1.5 and *p*-value<0.05 were considered DEGs. DEGs are displayed on a volcano plot. The 25 genes with the most significant differences in up- and down-regulation are shown on a heat map.

### 2.3 Functional enrichment analysis of significant DEGs

For gene set functional enrichment analysis, we used the Gene Ontology (GO) annotation in the R software package (version 3.1.0) as the background. The R software package clusterProfiler (version 3.14.3) was employed to map genes to the background and perform enrichment analysis, yielding gene set enrichment results. For KEGG Pathway analysis, we utilized the KEGG REST API (https://www.kegg.jp/kegg/rest/keggapi.html) to obtain the latest gene annotations of the gene annotations. Using this as a background, genes were mapped to the background collection, and enrichment analysis was conducted using clusterProfiler (version 3.14.3). We set the minimum gene set size to 5 and the maximum to 5000. Results were considered statistically significant with a *P*-value<0.05 and a FDR of<0.25 (18).

### 2.4 Protein–protein interaction network and module analysis

The STRING online tool (https://cn.string-db.org/) was used to construct the PPI network with a combined score > 0.4 (19). The PPI network was visualized using the Cytoscape application (20). Cytoscape’s CytoHubba plugins were utilized to filter important modules and core genes respectively. Five different algorithms Maximal Clique Centrality (MCC), Maximum Neighborhood Component (MNC), Degree (Deg), Edge Percolated Component (EPC) and EcCentricity were used for hub genes screening. Common genes identified by the five algorithms were considered reliable hub genes and demonstrated using a Venn diagram.

### 2.5 Hub genes verification and diagnosis model construction

GSE254880 was chosen as the validation set for hub genes. Single genetic diagnostic model was constructed using the pROC software package. The ROC curve was utilized to evaluate the discriminative ability of hub genes. Diagnostic value was quantified by calculating the area under the curve (AUC). An AUC greater than 0.7 is considered an ideal diagnostic indicator.

### 2.6 Homologous gene conversion

Gene conversion was conducted using the homologene tool in NCBI. We input the hub gene name and performed search to obtain homologous genes in other species via the NCBI website (https://www.ncbi.nlm.nih.gov/homologene).

### 2.7 Rat TBI samples

In animal experiments, twenty male wild-type Sprague-Dawley (SD) rats, aged six weeks, were purchased from Speford Biotechnology Company (Beijing, China). All rats were adaptively fed for two weeks before the experiment and fed a normal diet before and after the experiment. Ten rats randomly assigned to the control group, and the other 10 as the severe TBI model group. Rats were housed in a temperature-controlled environment (22-26°C, 40-70% humidity) with a 12-hour light-dark cycle. The rat traumatic brain injury model was constructed using the classic controlled cortical impact (CCI) method. Each rat was anesthetized via intraperitoneal injection of 3% pentobarbital sodium (0.2mL/100g), and then positioned on a stereotactic frame attached to a temperature controlled heating pad (37°C). The scalp was shaved, disinfected with 75% alcohol, and the scalp was incised on the left side of the midline of the brain. After exposing the skull, a 4mm craniotomy (3.0 mm AP and 2.0 mm ML to bregma) was performed on the cortex. A metal impactor (diameter=3mm) operated by pneumatic force impacts the brain at a speed of 3.5 m/s, reaching a depth of 1.0 mm below the dura mater and staying in the brain for 400 ms. Suture the wound after hemostasis and iodine disinfection. (21). After surgery, rats were placed in a constant temperature incubator at 30 °C and observed for 1 hour. Following this observation period, each rat was individually housed in cages with water containing recombinant food. Twenty-four hours after the completion of the modeling, 20 rats were euthanized by excessive anesthesia with pentobarbital sodium. Rats were initially perfused with physiological saline and then perfused with 4% paraformaldehyde. Immediately remove brain tissue after perfusion, and select the area from -2.12mm to -4.52mm of the anterior fontanelle as the experimental tissue based on the rat brain stereotaxic map (22). Optimal cutting temperature compound (OCT) was used to embed the brain tissue. Each sample was sliced into 20 tissue sections with a thickness of 30 μm. The animal experimental protocol was reviewed and approved by the Institutional Animal Care and Use Committee (IACUC) of Chinese PLA General Hospital (**Approval** ID: 2023-X19-128).

### 2.8 Immunofluorescence

Mouse anti-KDM5D (ab194288) MAb was purchased from Abcam, UK. Fluorescent secondary antibody (Bs-0295D-AF594) was purchased from Bioss, China. Anti-fluorescence attenuation mounting medium (containing DAPI, C190401) was purchased from YangGuangBio, China. All antibodies and reagents were used according to the manufacturer’s instructions. The immunofluorescence experiment was performed at the Lab Animal Center of Chinese PLA General Hospital. All of operations were performed in accordance with standard procedures (23). Fluorescence was observed under an Olympus BX53 fluorescence microscope (Olympus, Japan).

### 2.9 Immune infiltration analysis

The CIBERSORT package was used to evaluate the composition of immune and stromal cells in hippocampus (HIP) and prefrontal cortex (PCx) samples for immune cell infiltration analysis (24). We compared the expression of immune cells in HIP and PCx to study their role in TBI. A color bar graph displayed the proportion of each immune cell type in various samples. A t-test was used to compare the distribution differences of cells between disease and control groups, with a significance threshold set to *P*<0.05.

### 2.10 Expression analysis in pan-cancer

We downloaded the unified and standardized pan-cancer dataset TCGA TARGET GTEx (PANCAN, N=19131, G=60499) from the UCSC (https://xenabrowser.net/) database, and then extracted ENSG00000012817 (KDM5D) gene expression data from each sample. The samples were categorized into various sources, including Solid Tissue Normal, Primary Solid Tumor, Primary Tumor, Normal Tissue, Primary Blood Derived Cancer - Bone Marrow, and Primary Blood Derived Cancer - Peripheral Blood. Samples with an expression level of 0 were excluded, and each expression value underwent a log2 (x+0.001) transformation. Additionally, we also eliminated cancer types with less than three samples in a single cancer type. Finally, expression data for 32 cancer types were obtained.

### 2.11 Analysis of immune infiltration in pan-cancer

We downloaded the unified and standardized pan-cancer dataset TCGA TARGET GTEx (PANCAN, N=19131, G=60499) from the UCSC (https://xenabrowser.net/) database, and extracted ENSG00000012817 (KDM5D) gene expression data for each sample. The samples were sourced from Primary Blood Derived Cancer - Peripheral Blood (TCGA-LAML), Primary Tumor, TCGA-SKCM’s Metastatic, Primary Blood Derived Cancer - Bone Marrow, Primary Solid Tumor, and Recurrent Blood Derived Cancer - Bone Marrow samples. We filtered out samples with an expression level of 0, and applied a log2 (x+0.001) transformation to each expression value. Additionally, we mapped the the gene expression profile of each tumor to GeneSymbol. Using the R software package ESTIMATE (version 1.0.13, https://bioinformatics.mdanderson.org/public-software/estimate/), we calculated stromal, immune, and ESTIMATE scores for each patient, based on gene expression.

### 2.12 Statistical analysis

Statistical analysis and graphic design were performed using R software. All immunofluorescence experiments were repeated three or more times. Relative fluorescence intensity was calculated by Fiji (Image J), GraphPad Prism software (version 9.0) was used for fluorescence data analysis and graphing. A *P*-value<0.05 was considered statistically significant.

## 3 Result

### 3.1 Identification of DEGs of different brain areas in TBI and No-TBI

**Figure 1:**
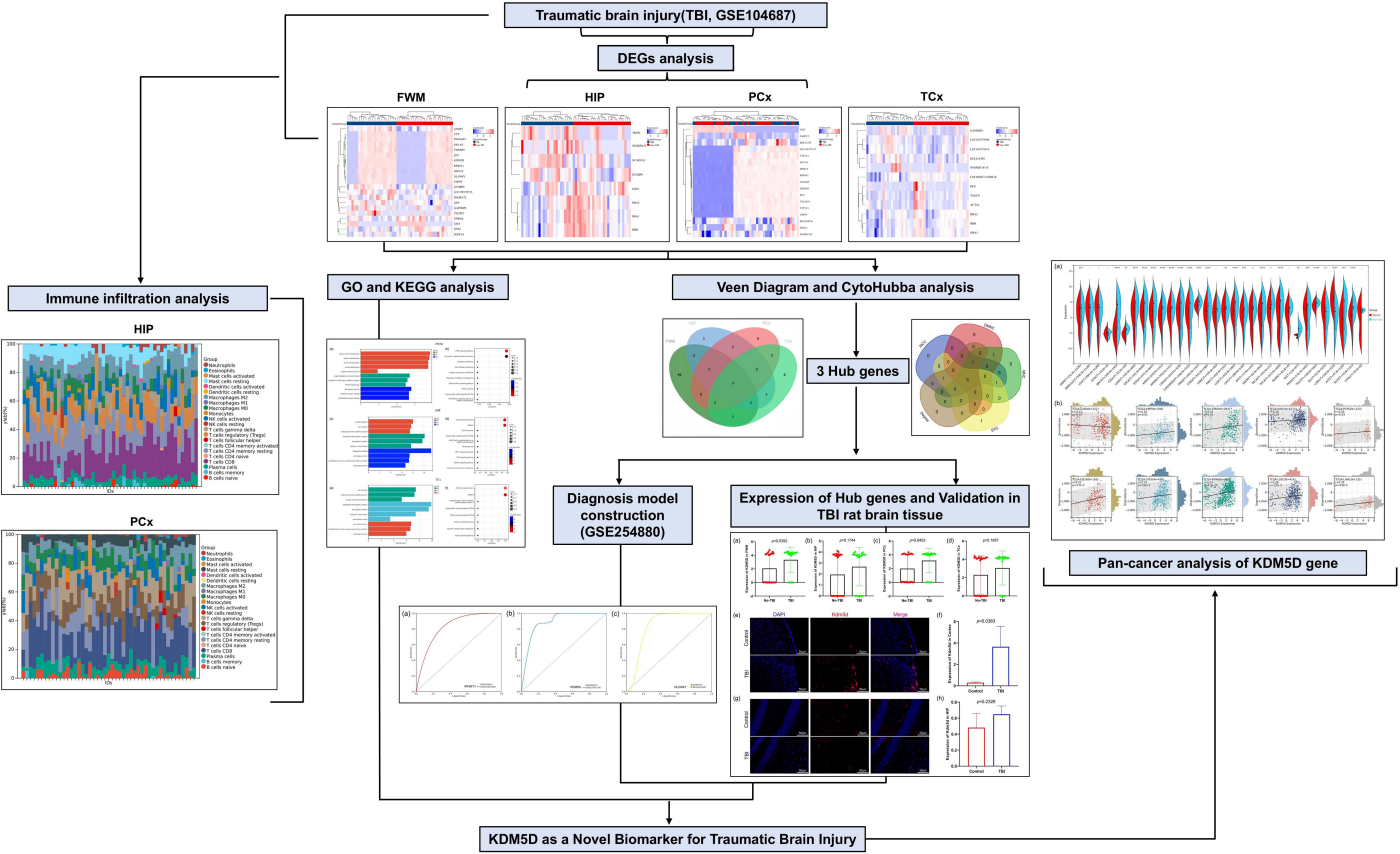
Research design flow chart.

**Figure 2:**
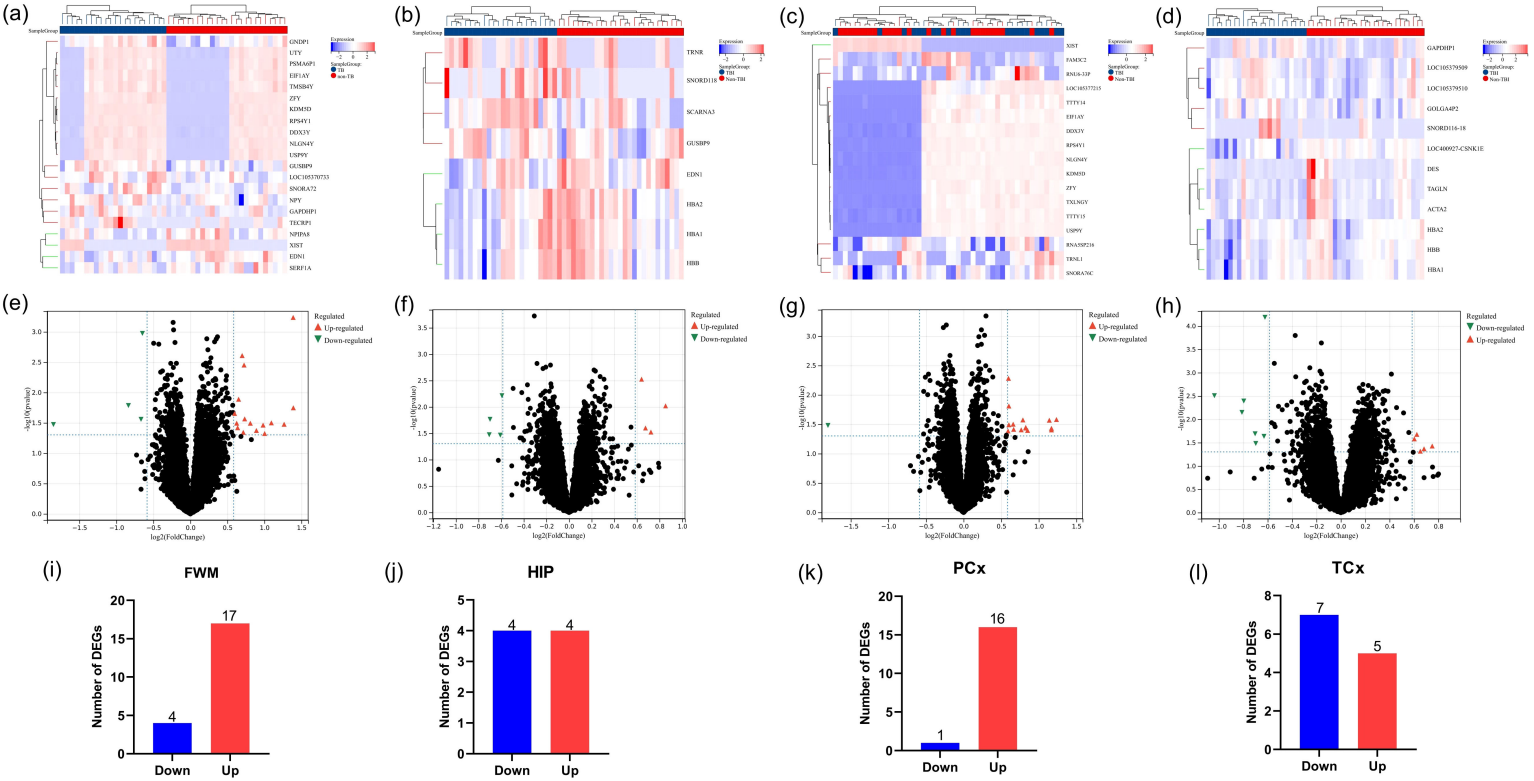
Identification of DEGs across four brain areas in TBI and non-TBI. **(a–d)** Cluster heat map displaying all DEGs in four brain regions between TBI and non-TBI. **(e–h)** Volcano map highlighting DEGs between TBI and non-TBI, with upregulated genes marked in light red and downregulated in light blue. The threshold was set to |log2FC (fold change)> 0, and p value < 0.05. **(i–l)** Bar charts illustrating DEGs in four brain areas of TBI and non-TBI.

The research flowchart of this study was shown in **Figure 1**. Samples from the GSE104687 dataset, downloaded from the GEO database, included transcriptome data from four brain regions: frontal white matter (FWM), hippocampus (HIP), prefrontal cortex (PCx) and temporal cortex (TCX). Differential gene analysis was conducted using the Limma package, revealing 21 significant DEGs (17 upregulated and 4 downregulated genes) in FWM between individuals with TBI and non-TBI (**Figures 2a, e, i**). Additionally, 8 significant DEGs (4 upregulated and 4 downregulated genes) were identified in HIP between TBI and non-TBI groups (**Figures 2b, f, j**). Furthermore, 17 DEGs (16 upregulated and 1 downregulated genes) were observed in in PCx between TBI and non-TBI groups (**Figures 2c, g, k**), and 12 DEGs (5 upregulated and 7 downregulated genes) were found in TCx between two groups (**Figures 2d, h, l**). Heat maps, volcano and bar charts depicted all DEGs in TBI and non-TBI, visualizing the findings of the cluster analysis. Please refer to **Supplementary Table S1** for a comprehensive list of DEGs.

### 3.2 Functional enrichment analysis of DEGs

**Figure 3:**
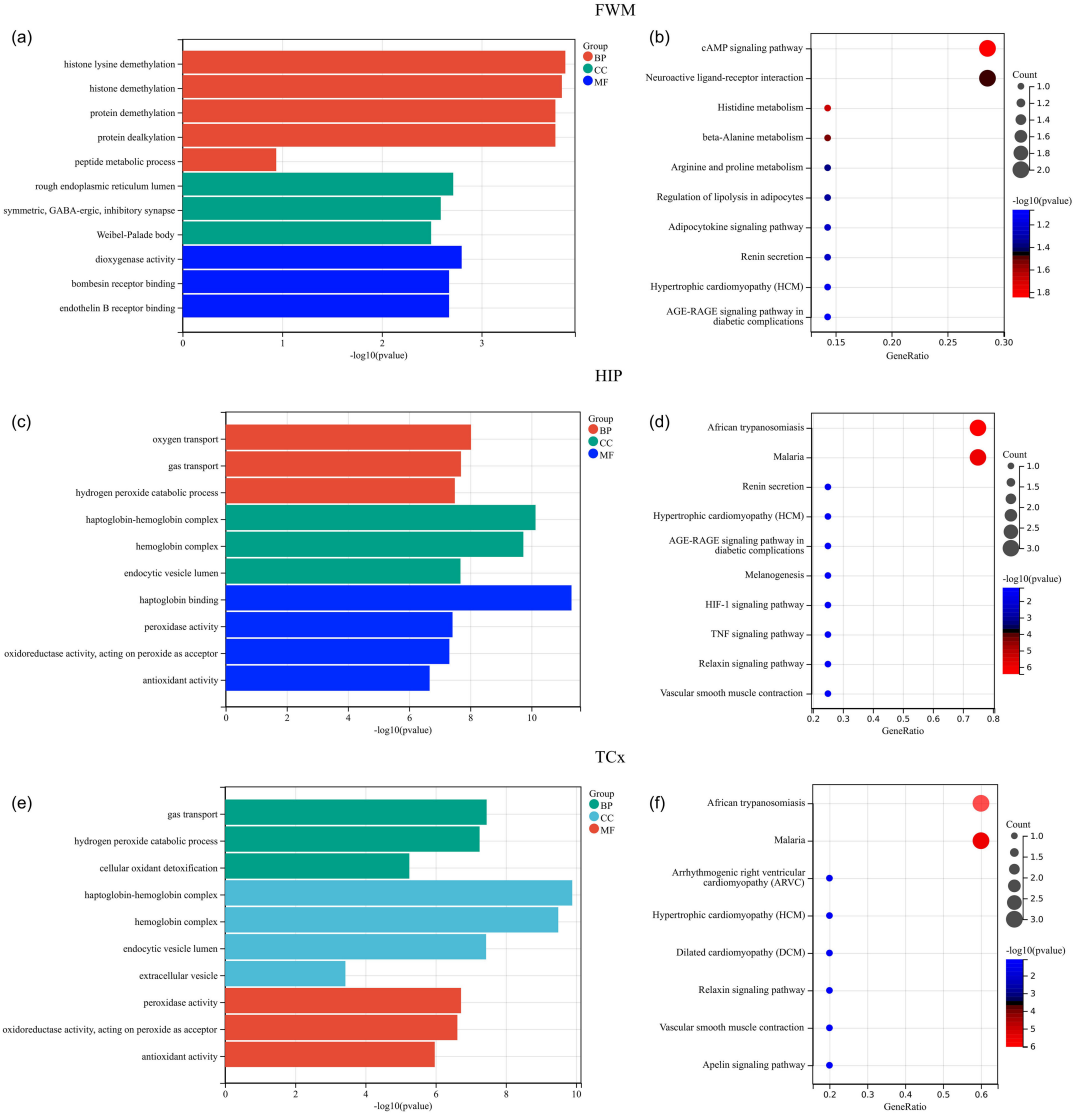
Functional enrichment analysis of DEGs. **(a, c, e)** GO analysis of DEGs in FWM, HIP and TCx, respectively. **(b, d, f)** KEGG pathway analysis of DEGs in FWM, HIP and TCx, respectively. P < 0.05.

To determine the function and pathways of the DEGs between TBI and non-TBI, we used “cluster profiler” to enrich GO and KEGG pathways. DEGs in four different brain regions were enriched and analyzed. Among FWM, GO terms mainly involve histone lysine demethylation, rough endoplasmic reticulum lumen, and dioxygenase activity (**Figure 3a**), KEGG pathways primarily involve the cAMP signaling pathway and neuroactive ligand-receptor interaction (**Figure 3b**). In HIP, GO terms mainly involve oxygen transport, haptoglobin-hemoglobin complex and haptoglobin binding (**Figure 3c**), KEGG pathways primarily involve African trypanosomiasis and Malaria (**Figure 3d**). In TCx, GO terms mainly involve gas transport, haptoglobin-hemoglobin complex and peroxidase activity (**Figure 3e**), KEGG pathways primarily involve African trypanosomiasis and Malaria (**Figure 3f**). Unfortunately, due to the large dispersion of DEGs in PCx, the enrichment analysis results were not available. These results suggest that intracellular signal transduction and cell signal transduction pathways are involved in the progression of the TBI. We showed the top ten results of GO and KEGG enrichment analysis. Please see **Supplementary Table S2** for full enrichment results.

### 3.3 PPI network analysis and identification of hub genes

**Figure 4:**
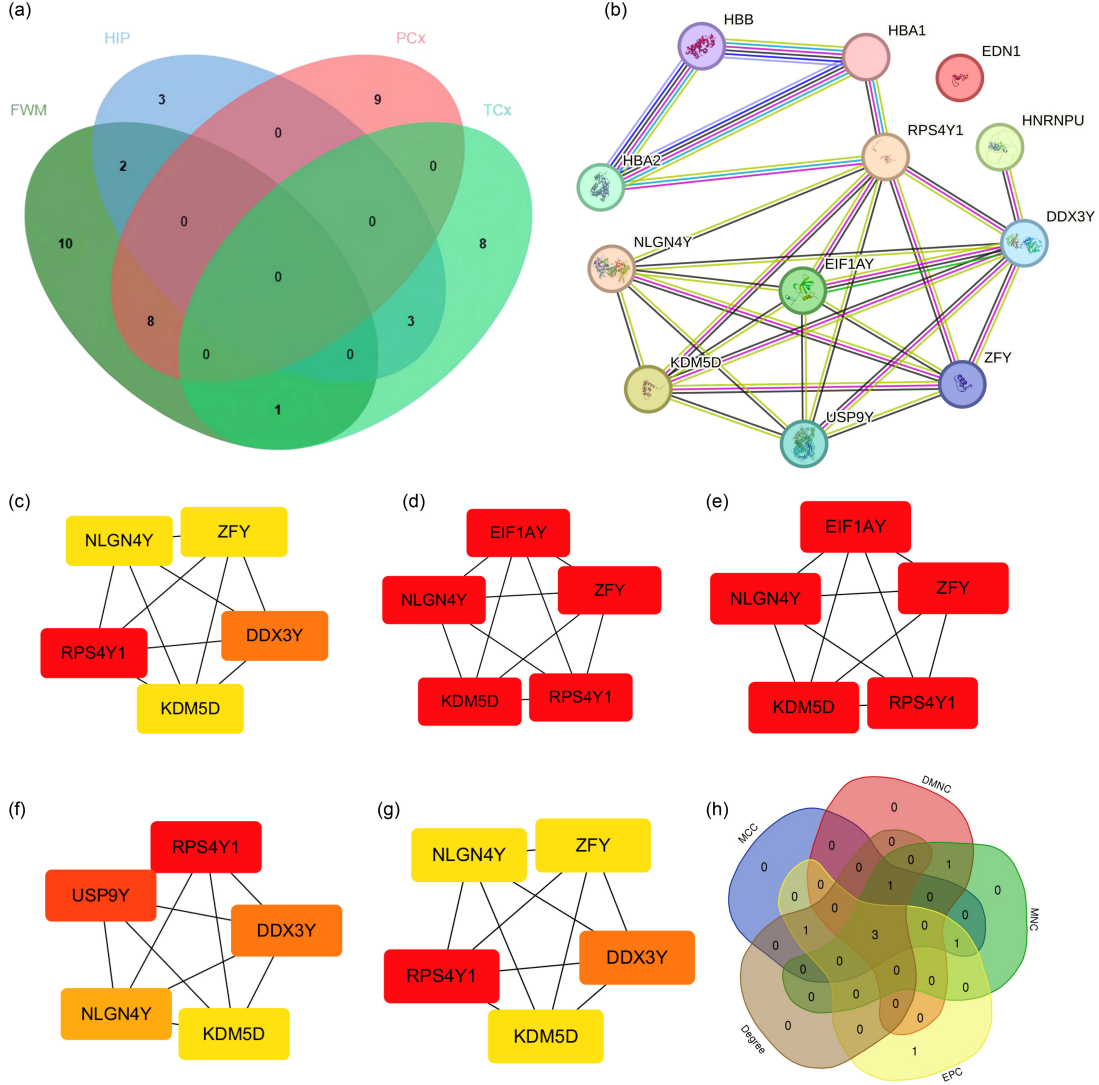
PPI network analysis and identification of hub genes. **(a)** Venn diagrams showing the distribution and overlap of DEGs across four brain regions. **(b)** PPI network highlighting communal hub genes. **(c–h)** Top scoring genes in various PPI network analysis.

To further identify key genes, Venn diagrams were used to show the distribution and overlap of DEGs in different brain regions. The results showed that FWM and HIP had 2 identical DEGs, FWM and PCx had 8 identical DEGs, and FWM and TCx had 1 identical DEGs, HIP and TCx have 3 identical DEGs, totaling 14 DEGs (**Figure 4a**). The STRING database was used to perform a PPI network analysis of the 14 communal DEGs to clarify the interactions between DEGs, revealing that 11 DEGs were closely related to each other (**Figures 4b**). In addition, the DEGs were ranked using the five algorithms DMNC, MCC, MNC, Degree and EPC in the cytoHubba plug-in, and the top 5 DEGs were obtained for each algorithm (**Figures 4c–g**). Subsequently, we analyzed the DEGs common to these five algorithms through a venn diagram, including RPS4Y1, KDM5D and NLGN4Y as hub genes (**Figure 4h**).

### 3.4 Diagnosis model construction of Hub genes

**Figure 5:**
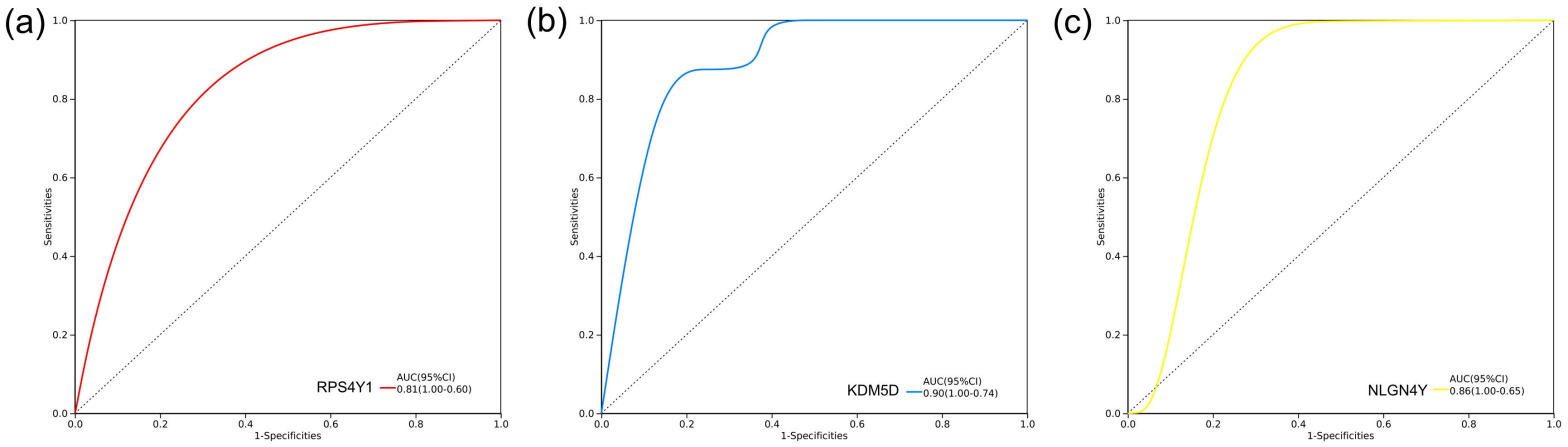
Diagnosis model construction of hub genes. ROC curve of diagnostic model based on three hub genes in GSE254880. **(a)** RPS4Y1 diagnostic model, AUC=0.81. **(b)** KDM5D diagnostic model, AUC=0.90. **(c)** NLGN4Y Diagnosis model of, AUC=0.86.

We completed the validation of the Hub genes in GSE254880. Further ROC curve analysis was conducted to verify the diagnostic value of Hub gene in GSE254880. In the single gene diagnostic model, the diagnostic value of the three hub genes RPS4Y1, KDM5D and NLGN4Y was meaningful, with KDM5D having the highest diagnostic value (AUC=0.90) (**Figures 5a–c**), suggesting that these three hub genes may be an effective indicator for detecting TBI.

### 3.5 Expression of KDM5D in TBI patients and TBI rat

To further support the involvement of the three hub genes in the pathogenesis of TBI, we intend to verify the protein expression of candidate genes. The literature review revealed that studies have shown that RPS4Y1 and NLGN4Y are related to TBI or brain injury (25, 26). However, there are no relevant studies on KDM5D and TBI.

**Figure 6:**
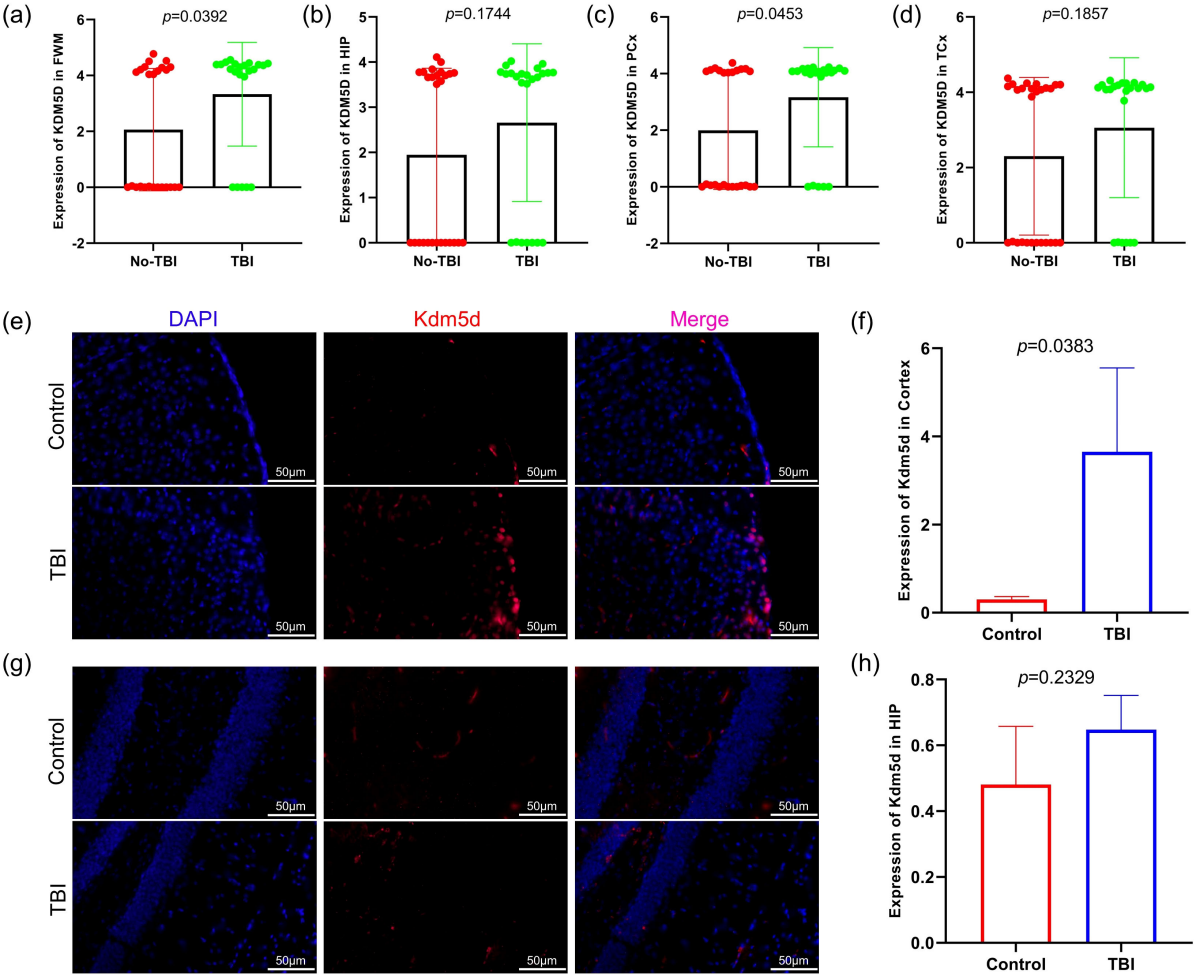
KDM5D expression in TBI patients and TBI rat. **(a–d)** Expression of KDM5D in four brain regions of TBI and non-TBI (GSE104687). **(e, f)** Expression of Kdm5d in cortical areas of TBI rats (p=0.0383). **(g, h)** Expression of Kdm5d in hippocampus areas of TBI rats (p=0.2329). Blue fluorescence indicates the nucleus, red fluorescence indicates Kdm5d and pink indicates the merge diagram. Scale bars: 50μm.

KDM5D is considered a novel marker and requires further investigation. We analyzed KDM5D mRNA expression in different brain regions and found significant differences in FWM (*P*=0.0392) and PCx (*P*=0.0453) (**Figures 6a–d**). Furthermore, we measured the expression of KDM5D protein in the brain tissue of TBI rats. Immunofluorescence staining showed that the expression of KDM5D protein increased in the cortex of TBI rats compared to control (**Figures 6e, f**), while there was no significant difference in the hippocampus no (**Figures 6g, h**).

### 3.6 Immune cell infiltration analysis

**Figure 7:**
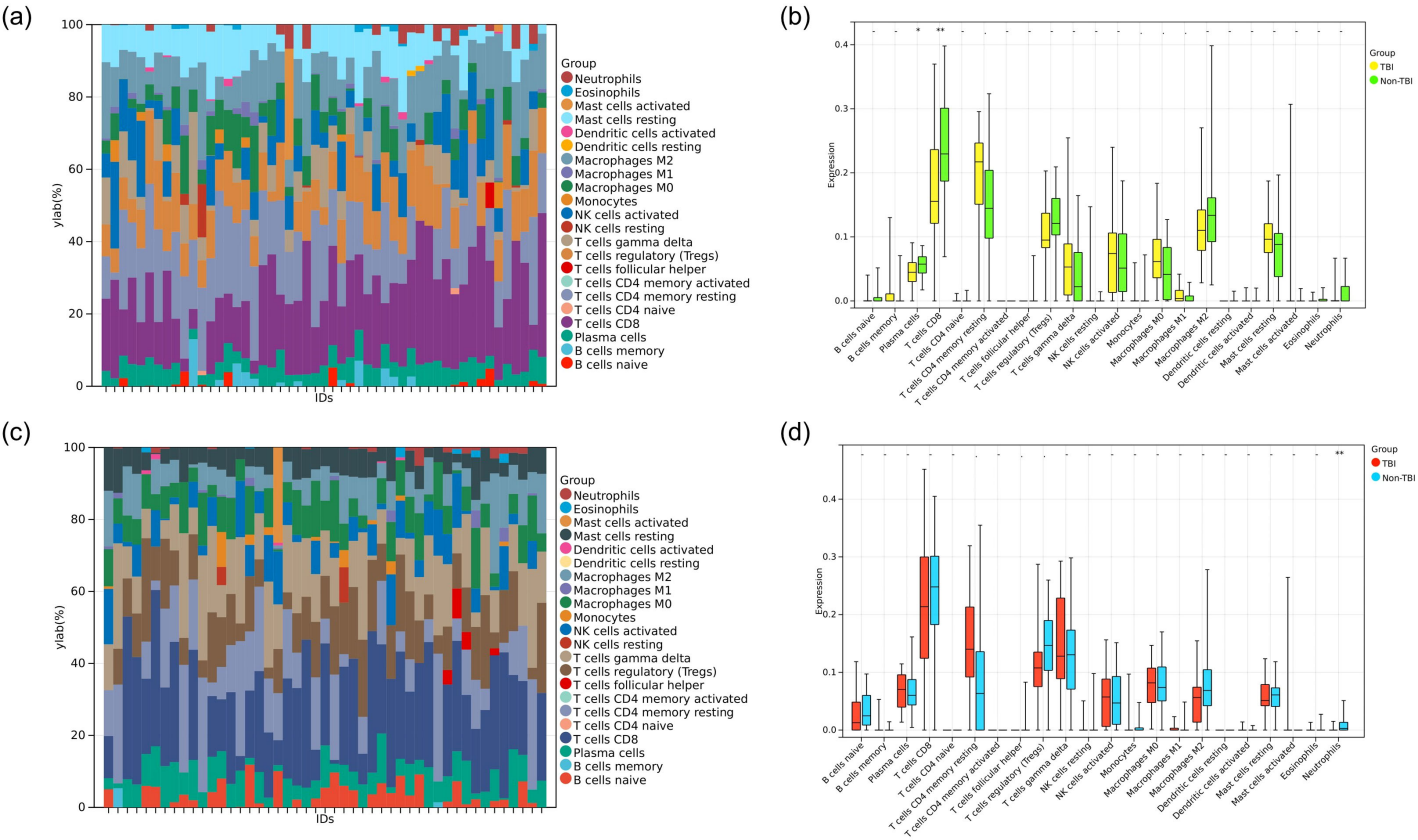
Analysis of immune cell infiltration. **(a)** Color bar plot illustrating the distribution of 22 immune cell types across various HIP samples. **(b)** Boxplot showing the expression profiles of two immune cell significant dysregulated in TBI compared to non-TBI. **(c)** Color bar plot illustrating the distribution of 22 immune cell types across PCx samples. **(d)** Boxplot showing the expression profile of one immune cell type significant dysregulated in TBI compared to non-TBI. *P<0.05, **P<0.01.

To investigate the role of immune cells in HIP and PCx of TBI, immune cell infiltration was analyzed in the HIP and PCx datasets using the CIBERSORT algorithm. The analysis results of the HIP dataset were shown in **Figure 7a**, and for PCx dataset in **Figure 7c**. These color bar graph clearly illustrate changes in the composition of immune cell subpopulations across the samples.

In the HIP dataset specifically, we examined the diversity in immune cell makeup between the TBI and non-TBI samples. This comparison revealed significant differences in the extent of infiltration of two immune cell types and highlighted the heterogeneity of immune responses associated with HIP. Specifically, plasma cells and T cells CD8 were downregulated in TBI (HIP) samples (**Figure 7b**).

Similarly, in the PCx dataset, the heterogeneity of immune cell composition was assessed between TBI and non-TBI samples. The results indicated significant differences in the infiltration of one type of immune cell Neutrophils, which were downregulated in TBI (PCx) samples (**Figure 7d**).

### 3.7 Analysis of KDM5D in pan-cancer

**Figure 8:**
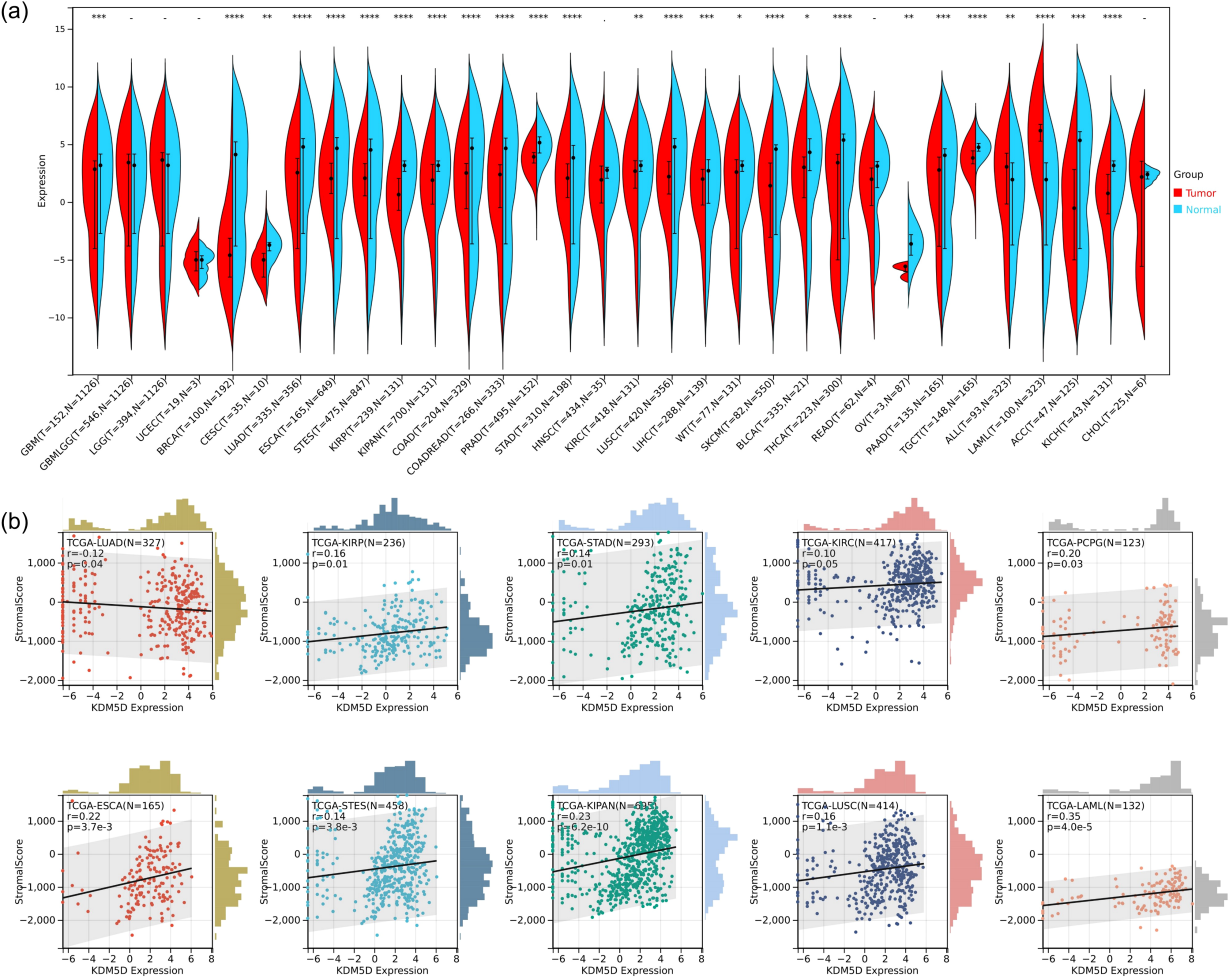
Analysis of KDM5D in pan-cancer. **(a)** Differences expression of KDM5D between tumors and normal tissues in the Cancer Genome Atlas (TCGA). *P<0.05, ** *P*<0.01 and *** *P*<0.001. **(b)** Immune correlation analysis of KDM5D across various immune infiltrates in human tumors. Positive values ​​indicate positive correlation, and negative values ​​indicate negative correlation.

One of the main hallmarks of TBI is the infiltration of different immune cell subpopulations. We speculate that this may also impact on tumor immune infiltration, so we aimed to further explore whether TBI-related KDM5D is involved in cancer progression. We used R software (version 3.6.4) to calculate the expression difference between normal and tumor samples across various cancer type, and used unpaired Wilcoxon Rank Sum and Signed Rank Tests for significance analysis. We observed significant upregulation in three tumors, including PAAD (Tumor: 0.92 ± 3.92, Normal: 0.81 ± 4.45, p=8.1e-4), ALL (Tumor: 1.29 ± 4.04, Normal: 0.39 ± 3.79, p=3.0e-3), and LAML (Tumor: 1.29 ± 4.04, Normal: 0.39 ± 3.79, p=3.0e-3). Conversely, we observed significant downregulation in 23 tumors, including GBM (Tumor: 0.57 ± 3.85, Normal: 1.38 ± 3.81, p=4.2e-4), BRCA(Tumor:-3.96 ± 3.00,Normal:0.82 ± 4.63,p=7.4e-18), CESC(Tumor:-5.14 ± 1.22,Normal:-3.94 ± 1.08,p=4.2e-3), LUAD (Tumor:0.69 ± 4.09,Normal:2.35 ± 4.21,p=8.1e-21), ESCA(Tumor:1.75 ± 2.39,Normal:2.04 ± 4.43,p=7.3e-9), STES(Tumor:1.44 ± 2.78,Normal:1.87 ± 4.41,p=4.4e-19),KIRP(Tumor:0.33 ± 2.58,Normal:2.19 ± 2.96,p=3.5e-18),KIPAN(Tumor:1.05 ± 3.03,Normal:2.19 ± 2.96,p=2.4e-9),COAD(Tumor:0.76 ± 3.68,Normal:1.57 ± 4.68,p=1.3e-10),COADREAD(Tumor:0.77 ± 3.58,Normal:1.57 ± 4.66,p=7.0e-12), PRAD(Tumor:3.72 ± 0.84,Normal:4.93 ± 0.96,p=8.7e-35), STAD(Tumor:1.27 ± 2.96,Normal:1.32 ± 4.32,p=4.4e-5), KIRC(Tumor:1.53 ± 3.20,Normal:2.19 ± 2.96,p=2.2e-3), LUSC(Tumor:1.53 ± 2.89,Normal:2.35 ± 4.21,p=1.0e-17), LIHC(Tumor:0.92 ± 2.89,Normal:1.30 ± 3.32,p=2.8e-4), WT(Tumor:0.30 ± 3.96,Normal:2.19 ± 2.96,p=0.04), SKCM(Tumor:0.23 ± 3.75,Normal:2.00 ± 4.13,p=1.7e-11), BLCA(Tumor:1.80 ± 3.06,Normal:2.54 ± 4.03,p=0.01),THCA(Tumor:0.14 ± 4.67,Normal:2.12 ± 4.73,p=1.4e-19), OV(Tumor:- 5.88 ± 0.54,Normal:-3.67 ± 1.20,p=9.6e-3), TGCT(Tumor:3.70 ± 1.04,Normal:4.78 ± 0.61,p=2.9e-24), ACC(Tumor:-0.82 ± 4.05, Normal:1.46 ± 5.17,p=2.3e-4), KICH(Tumor:0.33 ± 2.69,Normal:2.19 ± 2.96,p=9.1e-9) (**Figure 8a**).

Furthermore, we performed an analysis of immune infiltration of KDM5D in pan-cancer. We obtained immune infiltration scores for a total of 6389 tumor samples in 43 tumor types. We used the corr.test function of the R software package psych (version 2.1.6) to calculate the Pearson’s correlation coefficient between genes and immune infiltration scores in each tumor. In order to determine the significantly correlated immune infiltration score, we observed that the gene expression was significantly correlated with immune infiltration in 10 cancer types, with 9 showing a significant positive correlation, such as TCGA-ESCA (N=165, R=0.22, p=3.7e-3), TCGA-STES(N=458,R=0.14,p=3.8e-3),TCGA-KIRP(N=236,R=0.16,p=0.01), TCGA-KIPAN(N =695, R=0.23, p=6.2e-10), TCGA-STAD (N=293, R=0.14, p=0.01), TCGA-KIRC (N=417, R=0.10, p=0.05), TCGA -LUSC(N=414,R=0.16,p=1.1e-3), TCGA-LAML(N=132,R=0.35,p=4.0e-5), and TCGA-PCPG(N=123,R=0.20, p=0.03). There is 1 significant negative correlation, such as TCGA-LUAD (N=327, R=-0.12, p=0.04) (**Figure 8b**). Please see **Supplementary Table S3** for tumor full name and abbreviation.

## 4 Discussion

TBI has the highest incidence among common neurological diseases and poses a significant public health burden. TBI is recognized not only as an acute disease but also as a chronic one with long-term consequences, including an increased risk of delayed neurodegeneration. Although blood-based protein biomarkers have proven important in the assessment of TBI, most available assays are for research use only (27). Identifying more meaningful biomarkers is crucial for the diagnosis, treatment and prognosis of TBI. Transcript profiling is a promising tool for uncovering molecular mechanisms and biomarkers. Fortunately, we obtained transcriptome data of traumatic brain tissues from TBI patients in the GEO database. Damaged brain tissue not only reflects the pathological changes of the disease process (more clinical effects), but is also significant for the screening key genes. Additionally, for TBI patients, transcriptomic analysis and validation of brain tissue can more accurately describe the physio pathological changes of TBI.

In this study, we first identified the differential genes co-expressed by four brain areas of TBI and non-TBI patients through limma package, and obtained 44 DEGs (**Figure 2**, **Supplementary Table S1**). Subsequently, we explored the common biological functions and signaling pathways involved in these DEGs through GO and KEGG enrichment analysis. The results indicated that the mechanisms of TBI and non-TBI are mostly related to molecular modifications and signal transduction pathways, which could be the key mechanisms connecting the two.

The prominent enrichment of hemoglobin complex genes (HBA1/HBB) among our DEGs suggests an intriguing connection between erythrocyte extravasation and neuroinflammation post-TBI (**Figure 3**). Our co-expression analysis revealed strong correlations between HBA1 expression and microglial activation markers, supporting recent findings that hemoglobin breakdown products can activate TLR4 signaling in microglia (28). Clinically, we observed significant associations between HBB expression and both interleukin-6 (IL-6) levels and peripheral NOD-like Receptor (NLR) (29), suggesting these genes may serve as biomarkers for systemic inflammatory response after TBI. This aligns with Chen’s (30) demonstration of hemoglobin-driven inflammasome activation in subarachnoid hemorrhage models. TBI-induced brain damage manifests as excitotoxicity, neuroinflammation, cytokine damage, oxidative damage, and ultimately cell death (31). Studies have shown that it can prevent neuronal death and dysfunction in TBI by using of calpain inhibitors (AK295, SJA6017) and neurotrophic factors (NGF, BDNF) (32). Brain damage disrupts normal signaling pathways, leading to changes in brain function. Normally, in the central nervous system (CNS), signals are transmitted by various neurotransmitters such as gamma-aminobutyric acid (GABA), glutamate, glycine, norepinephrine, dopamine and serotonin. TBI may alter the levels of these neurotransmitters, ultimately disrupting the brain’s normal function (33). In summary, through enrichment analysis and previous studies, we believe that molecular modifications and signal transduction pathways are critical mechanisms of TBI.

To further identify key genes, we used Venn diagrams to show the distribution and overlap of DEGs in four brain regions (FWM, HIP, PCx, TCx), identifying 14 DEGs. We determined the protein interaction relationship of those DEGs through the STRING database, revealing that 11 DEGs were closely related (**Figures 4a, b**). However, we still could not obtain the Hub gene. Further analysis using the CytoHubba module algorithm analysis in Cytoscape identified 3 hub genes, including RPS4Y1, KDM5D and NLGN4Y (**Figures 4c–h**). Notably, validation results from external data sets (neuronal exosomes samples from serum) showed that RPS4Y1, KDM5D and NLGN4Y have high diagnostic value, with KDM5D having the highest diagnostic value (AUC=0.90) (**Figure 5**). This suggests that these hub genes can serve as biomarkers in both damaged brain tissue and serum, demonstrating high diagnostic significance.

Ribosomal protein S4 Y-linked 1 (RPS4Y1) is a member of the ribosomal protein S4E family and is encoded as ribosomal protein S4 located on chromosome p11.31. RPS4Y1 is ubiquitously expressed and plays an important role in correct development (34, 35). RPS4Y1 has been used as a marker for a variety of diseases, such as the disease resistance gene for corticosteroid combined with cyclosporine A treatment in Vogt-Koyanagi-Harada, and as a candidate gene in multiple sclerosis (36, 37). The latest single-cell transcriptome sequencing found that RPS4Y1 was up-regulated in four cell clusters: oligodendrocytes, microglia, astrocytes and neurons in post-traumatic brain tissue (26), which is consistent with our findings. Furthermore, there are studies suggesting that abnormal expression of RPS4Y1 may affect the signal transduction of signal transducer and activator of transcription 3 (STAT3). For instance, in preeclampsia induced hypertension, abnormal expression of RPS4Y1 impairs STAT3 signaling, thereby inhibiting the migration and invasion of trophoblast cells (34). STAT3 is known to have a protective effect on ischemic brain injury; however, it is still unclear which type of brain cell mediates this effect and through what mechanism. Research has shown that endothelial STAT3 may help prevent ischemic brain injury by protecting the endothelial function of cerebral blood vessels and the integrity of the blood-brain barrier (38). Therefore, it’s conceivable that abnormal expression of RPS4Y1 in TBI may cause dysfunction of cerebral arterial endothelial cells. For example, if RPS4Y1 overexpression damages the protective effect of STAT3 on cerebral blood vessels, it may exacerbate TBI. Identifying the potential mechanisms of RPS4Y1 and STAT3 action is a novel research direction for future studies.

Neuroligin 4Y (NLGN4Y) is a member of the neuroligin gene family located in Yq11 (39). Previous research found that NLGN4Y gene mutations play a crucial role in male homosexuality and autism. These mutations often lead to severe neurodevelopmental disorders (40, 41). In a report of pediatric traumatic brain injury, NLGN4Y may be involved in the convergence of sex differences with TBI, but no further studies have been conducted (42). In short, NLGN4Y as a gene related to neurodevelopment, deserves further exploration for its connection with TBI.

Lysine-specific demethylase 5D (KDM5D), a member of the KDM5 family, is a male-specific histone demethylase (43). Histone methylation and demethylation play crucial roles in gene regulation and biological events during disease development, including neurological disorders and multiple cancer types. Similar to other KDM5 family members, KDM5D enables dimethyl and trimethyl H3K4 demethylation. KDM5 is involved in the pathogenesis of various cancers by inhibiting the expression of certain genes at the transcriptional level (44–46). TBI can lead to cognitive decline and impaired memory consolidation, as the cerebral cortex and hippocampus are critical for controlling cognitive functions and social behavior (47, 48). In mice, *Kdm5d* is expressed in the cortex and hippocampus, which may be associated with the prevalence of multiple neurological and psychiatric disorders, such as multiple sclerosis, schizophrenia and autism (48). In our study, we found that compared with non-TBI, the expression of KDM5D in the four brain regions of TBI patients showed an upward trend, with significant difference in FWM and PCx (**Figures 6a–d**). Immunofluorescence experiments on brain tissue of TBI rats also showed that Kdm5d expression significantly increased in the cortical area, but no obvious difference in the hippocampus (**Figures 6e–h**). It is worth noting that Kdm5d is normally expressed in the cortex of the control rats. KDM5D is known to be physiologically expressed in the brain, particularly in the cortex, according to the Human Protein Atlas and functional studies (https://www.proteinatlas.org/). This basal expression likely reflects KDM5D’s normal roles in cortical functions, such as epigenetic regulation of gene expression and maintaining neuronal homeostasis (49, 50).The results of clinical trials and animal experiments are consistent, indicating that KDM5D expression changes after TBI occur mainly in cortical areas. Our key finding is the significant upregulation of KDM5D in the cortex following TBI, not its mere presence in the control brain. It is this marked increase in KDM5D expression in the context of TBI that points to its pathological role and diagnostic potential.

KDM5D encodes the histone H3 demethylase on K4, and H3K27me3 demethylase has been confirmed to activate microglia to produce inflammation through STAT and TLR signaling pathways (48, 51). This is similar to microglial activation after TBI (52). In addition, the epigenetic regulatory function of KDM5D, as a histone demethylase, suggests a potential mechanism for sustained changes in gene expression in microglia, potentially leading to prolonged activation (53). H3K27me3 was known to induce microglia to express pro-inflammatory genes through TLR (54), suggesting that KDM5D-mediated demethylation at specific gene loci in microglia could result in the sustained upregulation of pro-inflammatory mediators or the downregulation of genes responsible for resolving inflammation, thus contributing to a chronic activated state.

TLR signaling, particularly TLR4 which is known to be activated by various damage associated molecular patterns (DAMPs) released after TBI, is a potent inducer of pro-inflammatory responses in microglia. If KDM5D activation in microglia promotes TLR signaling, it could amplify the release of a range of DAMPs typically associated with TLR activation, such as High Mobility Group Box 1 (HMGB1), heat-shock proteins and extracellular ATP (55, 56). These DAMPs, in turn, can further propagate neuroinflammation and maintain prolonged microglial activation, potentially creating a feed-forward loop that sustains chronic inflammation in the TBI milieu (56–58).

Moreover, it’s conceivable that KDM5D-mediated epigenetic modifications could specifically influence the expression of genes encoding for certain types of DAMPs in microglia. For instance, if KDM5D demethylates and activates the promoters of genes encoding for pro-inflammatory cytokines or chemokines that can act as DAMPs (e.g., TNF-α, IL-1β, CCL2), this could result in a sustained release of these specific DAMPs, contributing to prolonged inflammation and microglial activation. Further research is warranted to investigate the precise DAMPs released by microglia upon KDM5D activation and TLR signaling in the context of TBI, and to elucidate the epigenetic mechanisms by which KDM5D regulates their expression. Identifying these specific DAMPs and the underlying mechanisms will be crucial for developing targeted therapeutic strategies to modulate microglial activation and neuroinflammation in TBI.

As mentioned before, we found that the expression of KDM5D in the cortex and hippocampus was completely inconsistent. We speculated that this may be related to immune cells. Through immune infiltration analysis of PCx and HIP brain regions in GSE104687, it was found that the expression of neutrophils in PCx was reduced in TBI (**Figure 7**). Previous studies have shown that TBI is accompanied by an increase in neutrophils, but most studies are limited to patients with mild and acute TBI or animal experiments. There are few studies on patients with severe or even fatal TBI (59). Recent reports suggest that neutrophil plasticity and crosstalk with other cells also complicate their function in TBI. Therefore, it is premature to conclude whether neutrophils are beneficial or detrimental in TBI. Neutrophils act in a well-defined and limited manner, as the protective or deleterious effects of neutrophils depend on the stage and type of injury, with which they interact. However, the molecular mechanisms of how the brain environment modulates neutrophil function and how neutrophil function affects brain injury and repair have not been well elucidated. More research is required to elucidate the complex roles of neutrophils at various stages of TBI and the underlying mechanisms. Future advancements may pave the way for the developing novel therapeutic strategies aimed at selectively eliminate the harmful effects of neutrophils, while preserving the beneficial impacts in TBI (60). Studies have showed that KDM5D is involved in neutrophil degranulation, activation, and neutrophil-mediated immune response, but the detailed molecular mechanism remain unreported (61). This finding may offer a promising direction for the diagnosing and prognosticating of TBI. Cancer is one of the most challenging global health concerns, sharing metabolic and risk factor characteristics with other diseases. Therefore, it is plausible that TBI biomarkers could also be used in tumor prediction strategies. Significant down-regulation of KDM5D was observed in 23 types of tumors, whereas up-regulation occurred in only 3 types of tumors (**Figure 8a**). Exploring KDM5D expression in cancer infiltration, we observed significant correlation with immune infiltration levels in 10 cancers (**Figure 8b**). This suggested a potentially adverse prognostic impact of effect.

At present, there is limited literature directly studying the role of KDM5D in TBI. However, based on its known functions in other diseases such as cancer and cardiovascular disease (CVD), further speculation on the potential mechanisms of KDM5D in TBI (such as cerebral vascular injury, endothelial dysfunction, or vascular inflammation) is particularly interesting. A proteomics study of CVD found that overexpression of KDM5D in patients with CVD resulted in reduced protein levels of their substrate (H3K4me3) and dysfunction of vascular endothelial cells (62). In TBI, cerebral vascular injury may lead to local ischemia and hypoxia, which in turn can cause changes in epigenetic modifications (63). Overexpression of KDM5D may exacerbate cerebral vascular injury by reducing H3K4me3 levels, inhibiting the expression of vascular repair and protective genes, such as endothelial nitric oxide synthase (eNOS) and phosphate and tension homology deleted on chromosome ten (PTEN). And KDM5D was found to be related to endothelial injury and neurological inflammation of cerebral microvessels (64, 65), although it did not directly indicate the role of KDM5D in TBI, which provides a new idea for our subsequent in-depth research.

While our findings highlight KDM5D as a promising diagnostic biomarker for TBI, we should acknowledge a significant limitation: the potential overlap in KDM5D expression with cancer. KDM5D is also expressed and dysregulated in various cancers, raising concerns about its specificity as a TBI-specific biomarker. Indeed, studies have reported altered KDM5D expression in a wide range of malignancies, with downregulation observed in clear cell renal cell carcinoma (66) and upregulation in colon cancer (67). This complex expression pattern in cancer underscores the challenge of relying solely on KDM5D to differentiate TBI from cancer, or even to diagnose TBI in patients with pre-existing or concurrent cancer.

In cancer, KDM5D alterations are often linked to epigenetic reprogramming and oncogenic signaling pathways, contributing to tumor development and progression (68). In contrast, in TBI, we propose that KDM5D upregulation is primarily triggered by neuroinflammation, cellular stress, and the cascade of secondary injury events following the initial trauma. This difference in upstream triggers could potentially lead to distinct downstream effects and temporal expression patterns of KDM5D in TBI versus cancer. To address the specificity limitations of KDM5D as a standalone TBI biomarker, we propose a multi-faceted diagnostic strategy. Firstly, combining KDM5D with established TBI biomarkers, such as Glial Fibrillary Acidic Protein (GFAP) and Neurofilament Light chain (NFL) (69), which exhibit high axonal specificity and are less likely to be influenced by cancer could significantly enhance diagnostic accuracy.

Secondly, incorporating cancer-specific markers, such as Carcinoembryonic Antigen (CEA) and Cancer Antigen 125 (CA125) (70), for individuals at risk or suspected of having cancer, could aid in differential diagnosis. Thirdly, integrating biomarker data with clinical findings and neuroimaging techniques would provide a more comprehensive diagnostic picture, especially in complex scenarios.

A particularly challenging clinical scenario arises when an individual presents with symptoms potentially indicative of both TBI and cancer, or when a patient with known cancer sustains a head injury. In such cases, relying solely on KDM5D might lead to misdiagnosis or an inaccurate assessment of injury severity or cancer progression, as KDM5D levels could be elevated due to either or both conditions. Our proposed multi-biomarker and multi-modal approach is specifically designed to mitigate this challenge and improve diagnostic accuracy in these complex clinical presentations.

## 5 Conclusion

Based on the bioinformatics analysis and animal experimental verification, we propose that KDM5D could serve as a potential function as a co-biomarker for predicting and diagnosing TBI and pan-cancer. Additionally, investigating KDM5D may lead to the development of novel serum markers, providing new indicators for clinical liquid biopsy and potentially enhancing strategies for TBI and cancer prevention.

## Data Availability

The original contributions presented in the study are included in the article/**Supplementary Material**. Further inquiries can be directed to the corresponding author.
